# Four cycles of paclitaxel and carboplatin as adjuvant treatment in early-stage ovarian cancer: a six-year experience of the Hellenic Cooperative Oncology Group

**DOI:** 10.1186/1471-2407-6-228

**Published:** 2006-09-25

**Authors:** Aristotle Bamias, Christos Papadimitriou, Eleni Efstathiou, Alexandros Rodolakis, Georgios Vlahos, Zannis Voulgaris, Georgios Bozas, Georgios Fountzilas, Gerassimos Aravantinos, Evagelia Razis, Dimitra Gika, Meletios A Dimopoulos

**Affiliations:** 1 Department of Clinical Therapeutics, Medical School, University of Athens, Athens, Greece; 2 First Department of Obstetrics and Gynaecology, Medical School, University of Athens, Athens, Greece; 3 Aristotle University of Thessaloniki, School of Medicine, Thessaloniki, Greece; 4 Third Medical Department, Agii Anargyri Hospital, Athens, Greece; 5 Hygia Hospital, Athens, Greece

## Abstract

**Background:**

Surgery can cure a significant percentage of ovarian carcinoma confined to the pelvis. Nevertheless, there is still a 10–50% recurrence rate. We administered paclitaxel/carboplatin as adjuvant treatment in early-stage ovarian carcinoma.

**Methods:**

Patients with stages Ia or Ib, Grade 2 or 3 and Ic to IIb (any grade) were included. Patients were treated with 4 cycles of Paclitaxel 175 mg/m^2 ^and Carboplatin [area under the curve (AUC) 6 (Calvert Formula)] every 3 weeks.

**Results:**

Sixty-nine patients with no residual disease following cytoreductive surgery and minimal or modified surgical staging were included in this analysis. Grade 3 or 4 neutropenia occured in 29.9% of patients, while neutropenic fever was reported in 4.5%. Neurotoxicity (all Grade 1 or 2) was reported in 50% of cases. Median follow-up was 62 months. 5-year overall survival (OS) and relapse-free survival (RFS) were: 87% (95% confidence intervals [CI]: 78–96) and 79% (95% CI: 69–89), respectively. Significantly fewer patients with stages Ic-IIb and tumor grade 2 or 3 achieved a 5-year RFS than patients with only one of these two factors (73% vs 92%, p = 0.03).

**Conclusion:**

Paclitaxel/Carboplatin chemotherapy is a safe and effective adjuvant treatment in early-stage ovarian carcinoma. Patients with stages Ic-IIb and tumor grade 2 or 3 may benefit from more extensive treatment.

## Background

Ovarian cancer is a common malignancy. In most cases the disease is diagnosed at an advanced stage. Nevertheless, about 30% of patients present with early-stage disease, i.e. confined to the pelvis without spread outside the gynaecological organs (FIGO stages I and II). Surgery alone can cure a significant percentage of these patients. Nevertheless, there is a 10–50% recurrence rate, depending on stage, differentiation and histological type [1-3]. Therefore, research has been focused in developing adjuvant treatment for patients in high-risk of relapse. Historically, radiotherapy, either as external beam abdominopelvic irradiation or as intraperitoneal (i.p.) ^32^P, was the first modality to be tested in early ovarian cancer. Although radiotherapy is effective in ovarian cancer, its use in this setting was not supported by the results of phase II and III studies [1,4-6], while its toxicity, especially of ^32^P, is substantial and cisplatin monotherapy was shown to be more effective and less toxic than ^32^P in one randomized study [5]. Therefore, systemic chemotherapy represents the adjuvant treatment, which has been tested in the most recent studies.

Few randomized trials have addressed the question whether adjuvant chemotherapy is of benefit in early ovarian cancer [5-10]. In the first three studies no survival benefit was shown [5-7], although in one study cisplatin produced a significant improvement in disease-free survival [6]. However, all these trials lacked statistical power to detect realistic but clinically relevant differences. Recently, two large randomized trials and a joint analysis of the results of the two trials (including 925 patients) have been published [8-10]. Treatment was not uniform since various combinations as well as single-agent platinum-based chemotherapy were acceptable, while the requirements of surgical staging, a factor, which is critical for prognosis but also for the interpretation of the results [4,6,8] were also different. In spite of these methodological differences, both trials showed a significant improvement of recurrence-free survival, while one of them as well as the joint analysis also showed a significant prolongation of 5-year survival by 8–9% [9,10].

In all previous studies most patients received cisplatin or carboplatin monotherapy or cisplatin-based combination chemotherapy without paclitaxel. The latter agent has recently emerged as an integral component of the standard first-line treatment for advanced ovarian cancer in combination with cisplatin or carboplatin, after the results of three randomized studies [11-13]. Nevertheless, its role in early stage disease had not until recently been studied.

Since 1996 most centers of the Hellenic Cooperative Group have been treating patients with stages I-IIb epithelial ovarian cancer with the combination carboplatin and paclitaxel after surgery. We are now reporting a retrospective analysis of the toxicity and efficacy of this combination in patients with a minimum follow up of 2 years.

## Methods

### Patients

All patients included in this analysis were managed in 4 centers of the Hellenic Cooperative Oncology Group (HECOG). This retrospective analysis was approved by the Scientific Committee of HECOG. The selection of patients was based on the following uniform criteria regarding the quality of surgery, administration of chemotherapy and follow up protocols. All patients had stages Ia or Ib, Grade II or III or any Grade Ic-IIb epithelial ovarian cancer. Patients with sex cord, germ cell or borderline tumors were not included. All patients had uniform surgical treatment and staging, which included at least: total abdominal hysterectomy (TAH) with bilateral salpingoophorectomy (BSO), inspection and palpation of all peritoneal surfaces and retroperitoneal area, biopsies of suspect lesions for metastases, infracolic omentectomy and peritoneal washings. Patients with other malignancies or WHO performance status > 2 were not included. All patients had normal bone marrow and liver function, serum creatinine ≤ 2 g/dl, no uncontrolled cardiac arrhythmias, heart or respiratory failure and negative post-surgery CT scan of the abdomen and pelvis. All surviving patients included in the analysis had a minimum follow-up of 2 years.

### Chemotherapy

Chemotherapy was initiated within 2 months from surgery apart from one case, where chemotherapy was started 4 months after surgery due to postsurgical complications. Paclitaxel at 175 mg/m^2 ^over 3 hours immediately followed by Carboplatin over 60 minutes were administered every 3 weeks. Four cycles of chemotherapy were administered. Carboplatin dose was based on the Calvert Formula to predict an AUC of 6 [14,15]. Chemotherapy was administered on schedule if neutrophil count was ≥ 1.5 × 10^9^/L (normal range: 2–7.5) and the platelet count ≥ 100 × 10^9^/L (normal range: 140–450). If absolute neutrophil count (ANC) was < 1.5 × 10^9^/L Granulocyte-colony stimulating factor (G-CSF) was administered until ANC recovery. If only one week delay was required, next chemotherapeutic courses were given every 4 weeks.

Apart from the addition of G-CSF no other dose modifications were applied for neutropenia with the exception of prolonged (more than 7 days with ANC < 0.5 × 10^9^/L) neutropenia, or febrile neutropenia in G-CSF supported patients. In these cases a 25% doses reduction for both drugs was performed. The following dose modifications were applied according to the nadir value of platelets: if platelets were between 25–49 × 10^9^/L a 25% dose reduction (all drugs) was made, while a 50% dose reduction was applied for platelet counts below 25 × 10^9^/L.

### Follow-up

Tumor assessment with chest X-ray, CTscan of the abdomen and pelvis and CA 125 was performed after completion of chemotherapy. Patients were then followed up every 3 months for the first two years, 6-monthly for the following 3 years and subsequently every year with physical examination (including gynaecological), CT scan of the abdomen and pelvis, chest X-ray and CA 125. Additional investigations were performed if clinically indicated.

### Statistical considerations

Overall survival (OS) and relapse-free survival (RFS) were calculated from the day of initiation of treatment until the date of last follow-up, death (for OS) or relapse (for RFS). Only radiological or clinical evidence of disease was considered as relapse, while the sole CA125 elevation above the upper normal limit was not. Survival curves were produced with the Kaplan Meier method and survival functions were compared across different groups with the log-rank test [16]. Missing values in any of the studied variables were omitted from the analyses. All analyses were performed using the SPSS statistical software (SPSS for Windows, version 10, SPSS Inc., Chicago, IL). Throughout the analysis a level of 5% was used to denote statistical significance except as indicated.

## Results

### Patients

Sixty-nine consecutive patients with stage I-IIb epithelial ovarian cancer, treated with chemotherapy between May 1996 and September 2002, who fulfilled the criteria mentioned above, have been included in this analysis. Baseline characteristics are shown in Table 1. All patients underwent optimal surgical debulking (i.e. no residual disease after cytoreductive surgery). Oophorectomy was performed in two patients who had undergone hysterectomy in the past. Fertility-preserving surgery (unilateral oophorectomy + omentectomy) was performed in one patient who had a successful pregnancy following the completion of chemotherapy. The predominant histological types were serous (29%) and endometrioid adenocarcinomas (26.1%), while "other" carcinomas included a malignant Brenner tumor, a transitional cell carcinoma and a mixed (mucinous + endometrioid) adenocarcinoma. Baseline CA125 level was missing in 10 patients. Twenty of the 59 patients (34%) with available baseline CA125 had elevated levels (> 37 U/ml). The median pretreatment CA125 in patients with elevated values was 66.5 U/ml (range 37–224). In 19 cases CA125 value normalized during treatment. In the remaining case the patient refused to provide further CA125 evaluation.

### Chemotherapy

Four cycles of Paclitaxel/Carboplatin were administered in all but 4 cases: 1 cycle of chemotherapy was administered in 2 cases (one patient developed renal failure after the first cycle of chemotherapy and the other refused further treatment), 3 cycles in 1 case (the patient developed pulmonary edema not related to chemotherapy after the third cycle and did not receive the fourth cycle) and paclitaxel was omitted from the 3 final cycles in one case of anaphylaxis after the first administration of paclitaxel. Toxicity data were not available in 2 cases. Alopecia was universal. The remaining toxicities are shown in Table 2. There were 20 cases (29.9%) of grade 3/4 neutropenia, which was complicated with fever in only 3 cases (4.5%). Non hematological Grade 3 or 4 toxicities were reported in only 4 cases: 1 renal, 1 vomiting, 1 liver and 1 allergy. The most frequent non-hematological toxicity was neurotoxicity, which was reported in 33 patients (49.3%). Allergic reaction was reported in 1 case with anaphylaxis (1.5%). There were no treatment related deaths.

### Relapse-free and overall survival

Median follow-up at the time of analysis was 62 months (22–97.5). During follow-up 14 patients relapsed and 7 died due to progression of their disease. All but two relapses occurred in patients with stages Ic and II and tumors of grade 2 or 3. Intraabdominal disease was the site of relapse in all but two cases who also had supradiaphragmatic disease. All but one relapses occurred within 5 years from the initiation of chemotherapy.

**Figure 1:**
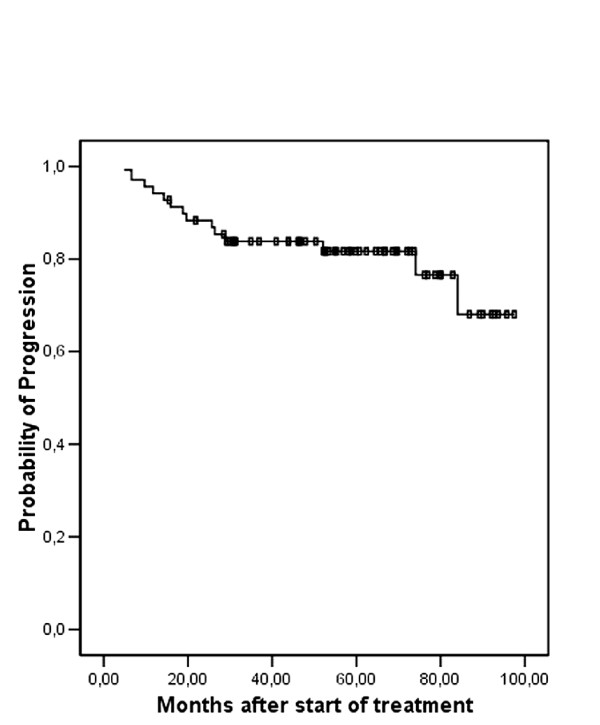
Relapse-free survival of 69 patients treated with paclitaxel/carboplatin for  early-stage (FIGO stages I-IIb) ovarian carcinoma.

**Figure 2:**
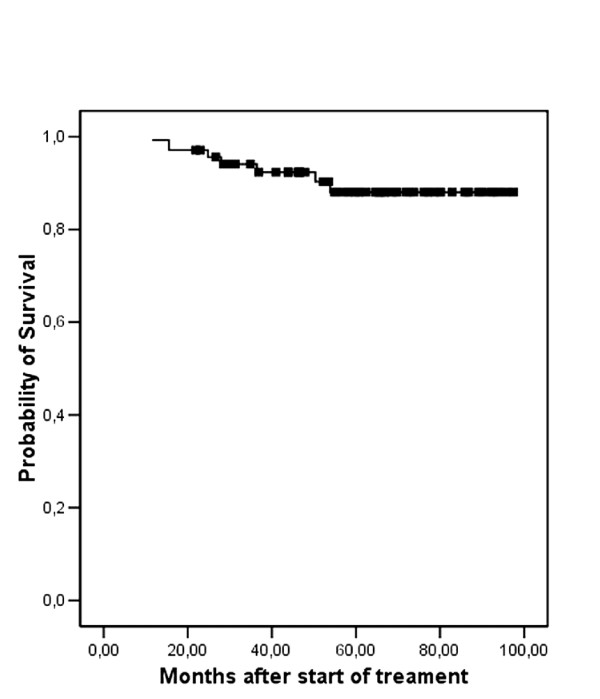
Overall survival of 69 patients treated with paclitaxel/carboplatin for early-stage (FIGO stages I-IIb) ovarian carcinoma.

**Figure 3:**
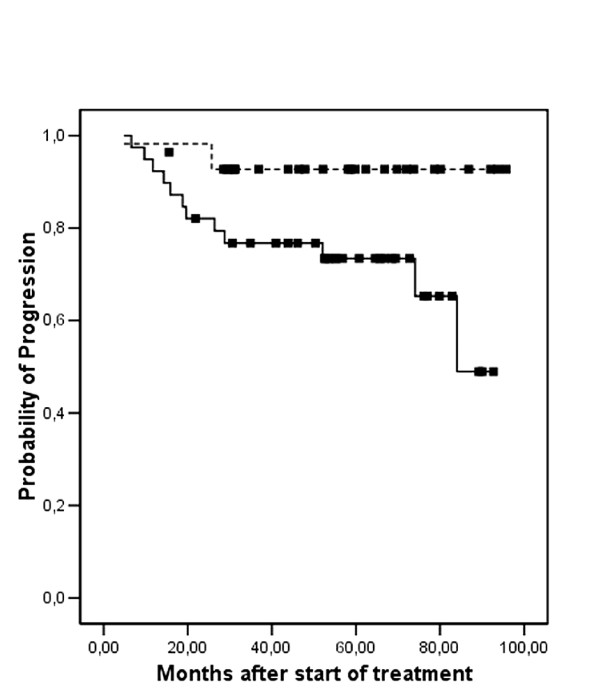
Relapse-free survival of patients with stages Ia/Ib OR tumor grade 1 (--)  vs. stages Ic/IIa/IIb AND tumor grade 2/3 (-).

**Figure 4:**
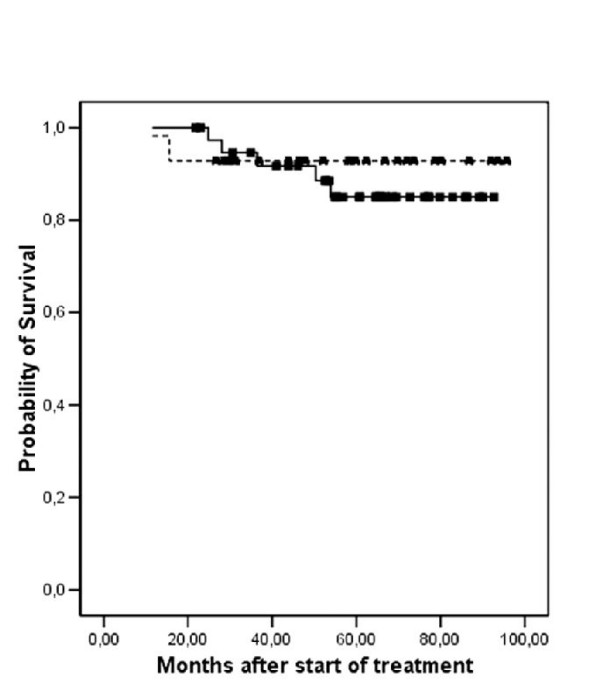
Overall survival of patients with stages Ia/Ib OR tumor grade 1 (--) vs. stages Ic/IIa/IIb AND tumor grade 2/3 (-).

Five-year RFS of the whole population was 79% (95% CI: 69–89) (figure 1) and 5-year OS was 87% (95% CI: 78–96) (figure 2). Table 3 shows the respective values according to grade and stage. RFS or OS were worse for patients with stages Ic and II and for patients with tumor grade 2 or 3 but these differences were not statistically significant. In addition, there were no significant differences among different histological types (p = 0.32) or according to baseline CA125 (normal vs. abnormal, p = 0.97). When stage and grade were combined, patients with stages Ic and II AND grade 2 or 3 had a significantly worse RFS compared to the remaining patients (5-year RFS 73% vs. 92%, p = 0.03) (Figure 3), while OS survival was also worse but this difference was not statistically significant (5-year OS 84% vs. 93%, p = 0.58) (Figure 4).

## Discussion

The role of systemic chemotherapy in ovarian cancer confined to the pelvis (FIGO stages I and II) remains controversial. Data from recent randomized studies [8-10] and systemic reviews of adjuvant treatments used in these patients [17] suggest that there is a benefit from platinum-based chemotherapy. Nevertheless, several important issues, i.e. the role of taxanes, the number of cycles and the selection of patients most likely to benefit from this treatment, remain unanswered.

We used 4 cycles of carboplatin/paclitaxel as adjuvant treatment in optimally debulked patients with I-IIb ovarian carcinoma. We did not include patients with stages Ia, Ib and Grade 1, since they have an excellent prognosis without adjuvant therapy [4,17]. There are no published data on the efficacy and tolerability of this regimen in this setting, since it has only recently been accepted as the standard in more advanced ovarian cancer [11,12]. By virtue of the Goldie hypothesis, this regimen should work better in earlier stages. For this reason we believe that the information reported here is of interest, since it is likely that this combination will also be extensively studied in early-stage ovarian cancer. This speculation is supported by the fact that this regimen has been used in a recent randomized trial [18]. The number of cycles was chosen because of our previous experience, which showed excellent toxicity profile in patients with bladder cancer [19]. Additionally, there were no data regarding the optimal number of cycles in early ovarian cancer at the time of the initiation of this treatment. In concert with our previous experience treatment was well tolerated. Grade 3 and 4 toxicities were rare except from neutropenia, which, however, had no clinical sequelae in all but 3 cases. Neurotoxicity was also frequent (50% of cases) but no grade 3 or 4 toxicities were reported.

The 5-year RFS of 79% and OS of 87% in our study are comparable to those reported by others for stages I and II disease receiving adjuvant platinum-based chemotherapy (Table 4). Baseline tumor stage, grade and histology of our patients are similar to those included in other studies. Although the retrospective nature of our study is a limitation in the interpretation of our findings and direct comparisons can only be validated through randomized trials, our results indicate that 4 cycles of carboplatin/paclitaxel is not an inferior treatment compared to previously used combinations or monotherapy (Table 4).

In spite of the apparent efficacy of our regimen, the following factors should be taken into consideration for the interpretation of our results: surgical staging, type of chemotherapy and number of administered cycles of chemotherapy. The importance of surgical staging in the prognosis and management of early-stage ovarian cancer has long been recognized [6,21,22]. This was also shown in the recent EORTC trial where 4 different categories of surgical staging (optimal, modified, minimal and inadequate) were clearly defined [8]. Although the quality of surgical staging was not predefined according to this system, the selection of our patients required at least minimal surgical staging (TAH and BSO, inspection and palpation of all peritoneal surfaces and the retroperitoneal area, biopsies of all suspect lesions, peritoneal washings and omentectomy). Information about the quality of surgery was based on the operative notes, which were available in 60 of the 69 cases. In the remaining 9 cases details of surgery was obtained by the treating physician. In 61 cases surgical staging was minimal while in the 8 cases of lymph node sampling it could be categorized as modified. Therefore, inaccurate staging cannot be excluded in some cases since about 17% of presumed stage I inadequately staged patients are upstaged to stage III after proper surgical staging [23]. Nevertheless, this type of surgery reflects the surgery performed in most randomized trials studying adjuvant treatment in early stage ovarian cancer [17], while suboptimal surgical staging is a well recognized problem in early ovarian cancer [24,25]. Even in the EORTC trial most patients underwent modified or minimal surgical staging [8]. In the same study there was no prognostic significance of surgical staging among patients who received chemotherapy. For the above reasons we believe that our results are clinically relevant.

The use of paclitaxel/platinum combination as opposed to the less neurotoxic platinum monotherapy in early ovarian cancer remains an unresolved issue. Monotherapy or treatment with cisplatin/cyclophosphamide have been mainly used in most studies up to now [1,5,8,9,20]. The combination of paclitaxel with cisplatin or carboplatin has been proposed as the standard in advanced disease but a recent randomized study showed no survival advantage over carboplatin monotherapy [26]. Therefore, it is important to determine any possible advantage of paclitaxel/platinum combination compared to platinum monotherapy taking into consideration the high percentages of alopecia and neurotoxicity associated. In contrast, the lower number of cycles of paclitaxel/carboplatin may be more preferable to the patients than the 6 cycles usually administered in the other studies. Since early stage ovarian cancer represents a prognostically diverse population, future studies should be focused on identifying the optimal treatment for the various subgroups of patients. Until then the standard chemotherapy in early stage remains debatable, although it should be stressed that paclitaxel/carboplatin has been used as the reference regimen in the two more recent randomized studies in early stage ovarian cancer [18,19].

The optimal number of cycles of chemotherapy in early-stage ovarian cancer has not been adequately addressed. Six cycles have been used in most studies [1,5,8,9]. This issue has been addressed in a randomized trial, reported in abstract form. Three cycles of paclitaxel/carboplatin were compared with 6 cycles of the same treatment [18]. This study showed no significant difference in 5-year RFS (73% vs. 81%), between the two arms. Anemia, granulocytopenia, and neurotoxicity were significantly higher with 6 cycles of treatment. These results indicate that 6 cycles of chemotherapy are not necessary for early ovarian cancer. This speculation is also supported by our results, since 4 cycles of chemotherapy achieved a 5-year RFS of 79%, similar to that reported after 6 cycles of treatment in the study by Young et al. Nevertheless, it cannot be excluded that some patients might benefit from more chemotherapy. We attempted to address this issue by identifying subgroups with inferior prognosis. Patients with early-stage ovarian cancer represent a heterogeneous group regarding their prognosis [2-4]. FIGO stage and tumor grade are the most consistent prognostic factors identified in previous studies [2-4,8]. We did not find any prognostic significance of these or any other baseline characteristic in our study. This could be due to the relatively small number of patients included in this analysis. Nevertheless, it should be stressed that tumor stage was not associated with prognosis in a recent randomized trial [8] as well as a metaanalysis of 1500 cases of stage I ovarian cancer [27]. The combination of tumor stage and grade identified a subgroup (stages Ic or II AND grade 2 or 3), which had a significantly inferior 5-year RFS compared with patients with only one of these factors (73% vs. 92%). Although the number of our patients is relatively small for subgroup analysis, we believe that these patients may represent a target population, who might benefit from the administration of 6 cycles of chemotherapy.

## Conclusion

Four cycles of paclitaxel/carboplatin chemotherapy represent an effective and well tolerated adjuvant treatment in this cohort of patients with stages I-IIb epithelial ovarian carcinoma, who underwent minimal or modified surgical staging. Patients with stages Ic/II and tumor grade 2/3 had worse progression-free survival than patients with only one of these factors and might benefit from the administration of more cycles of chemotherapy.

## Competing interests

The author(s) declare that they have no competing interests.

## Authors' contributions

AB contributed in the acquisition of data and the statistical analysis and drafted the manuscript. CP, EE, AR, GV and ZV were involved in the acquisition of data (treatment of the patients with surgery and chemotherapy) and critically revised the manuscript. GB and DG contributed in the acquisition of data and the statistical analysis. GA, GF and ER made substantial contribution in the conception and design of the study and the acquisition of data. MAD made substantial contribution in the conception and design of the study and the acquisition of data and critically revised the manuscript. All authors read and approved the final manuscript.

## Pre-publication history

The pre-publication history for this paper can be accessed here:



## Figures and Tables

**Table 1:** Patients characteristics at the initiation of chemotherapy

Characteristic	Pts	(%)
Age		
Median (range)	57 (24–78)	

Stage		
Ia	15	(21.8)
Ib	1	(1.4)
Ic	47	(68.2)
IIa	5	(7.2)
IIb	1	(1.4)

Histology		
Serous	20	(29)
Endometrioid	18	(26.1)
Mucinous	10	(14.5)
Clear cell	11	(15.9)
Poorly differentiated	5	(7.2)
Adenocarcinoma (not specified)	2	(2.9)
Other	3	(4.4)

Grade (n = 66)		
1	12	(18.2)
2	36	(54.5)
3	18	(27.3)
Missing	3	

Type of surgery		
TAH+BSO	7	(10.1)
TAH+BSO+omentectomy	51	(74)
TAH+BSO+omentectomy+LND	8	(11.6)
BSO+omentectomy	2	(2.9)
BSO	1	(1.4)

Baseline CA125 (n = 59)		
Elevated	20	(34)
Normal	39	(66)

TAH: total abdominal hysterectomy; BSO: bilateral salpingoophorectomy; LND: lymphadenectomy

**Table 2:** Worst toxicity of 67 patients who received Paclitaxel/Carboplatin

Toxicity	0 n(%)	1 n(%)	2 n(%)	3 n(%)	4 n(%)
Anemia	31 (46.3)	29 (43.3)	7 (10.4)	0 (0)	0 (0)
Neutropenia	21 (31.3)	6 (9)	20 (29.8)	14 (20.9)	6 (9)
Thrombocytopenia	64 (95.5)	2 (3)	1 (1.5)	0 (0)	0 (0)
N+V	45 (67.2)	18 (26.8)	3 (4.5)	1 (1.5)	0 (0)
Renal	66 (98.5)	0 (0)	0 (0)	0 (0)	1(1.5)
Diarrhea	66 (98.5)	1 (1.5)	0 (0)	0 (0)	0(0)
Stomatitis	65 (97)	0 (0)	2 (3)	0 (0)	0 (0)
Neurotoxicity	34 (50.7)	27 (40.3)	6 (9)	0 (0)	0 (0)
Liver	66 (98.5)	0 (0)	0 (0)	1 (1.5)	0 (0)
Allergy	66 (98.5)	0 (0)	0 (0)	0 (0)	1 (1.5)
Neutropenic fever	64 (95.5)	N/A	N/A	3 (4.5)	0 (0)

**Table 3:** Five-year overall survival and relapse-free survival of 69 patients who received Paclitaxel/Carboplatin

	5y OS (95% CI)	P*	5y RFS (95% CI)	P*
All patients (n = 69)	87% (78–96) no of deaths: 7		79% (69–89) no of relapses: 14	

Stage		.81		.28
Ia, Ib (n = 16)	94% (82–100) No of deaths: 1		93% (79–100) No of relapses: 1	
Ic, II (n = 53)	86% (75–97) No of deaths: 6		78% (66–90) No of relapses: 13	

Grade		.88		.30
1 (n = 12)	92% (76–100) No of deaths: 1		92% (76–100) No of relapses: 1	
2,3 (n = 54)	86% (75–97) No of deaths: 6		78% (66–90) No of relapses: 13	

Ia/Ib OR grade 1 (n = 28)	93% (83–100) No of deaths: 2	.58	92% (82–100) No of relapses: 2	.03
Ic/II AND grade2/3 (n = 39)	84% (71–97) No of deaths: 5		73%(58–88) No of relapses: 12	

*p values estimated with log rank test

**Table 4:** Published studies of adjuvant chemotherapy in early stage ovarian cancer

Study	Type of study	Treatment	Pts	5y RFS	5y OS
Young, 1990^6^	Prospective, randomized	Melphalan ^32^P	68 73	80% 80%	81% 78%
Vergote, 1992^1^	Prospective, randomized	Cisplatin ^32^P	171 169	81% 83%	75% 81%
Bolis, 1995^5^	Prospective, randomized	Cisplatin ^32^P	82 79	85% 65%	81% 79%
Young, 1999^20^	Prospective, randomized	Cyclo/Cisplatin ^32^P	107 98	77% 66%	84% 76%
Trope, 2000^7^	Prospective, randomized	Carboplatin No treatment	81 81	70% 71%	86% 85%
Trimbos, 2003^8^	Prospective, randomized	Platinum-based No treatment	224 224	76%* 68%	85% 78%
ICON1, 2003^9^	Prospective, randomized	Platinum-based No treatment	241 236	73%^@ ^62%	79%^# ^70%
Present study	Retrospective, nonrandomized	Paclitaxel/Carboplatin	69	79%	87%

RFS: relapse-free survival; OS: overall survival; *: p = 0.02; @: p = 0.01; #: p = 0.03
